# Bioinspired polymer microstructures for directional transport of oily liquids

**DOI:** 10.1098/rsos.160849

**Published:** 2017-03-15

**Authors:** C. Plamadeala, F. Hischen, R. Friesenecker, R. Wollhofen, J. Jacak, G. Buchberger, E. Heiss, T.A. Klar, W. Baumgartner, J. Heitz

**Affiliations:** 1 Institute of Applied Physics, Johannes Kepler University Linz, 4040 Linz, Austria; 2 Institute of Biomedical Mechatronics, Johannes Kepler University Linz, 4040 Linz, Austria; 3 Tyrolean State Museum, 6020 Innsbruck, Austria

**Keywords:** biomimetics, two-photon lithography, directional transport

## Abstract

Nature has always served as an inspiration for scientists, helping them to solve a large diversity of technical problems. In our case, we are interested in the directional transport of oily liquids and as a model for this application we used the flat bug *Dysodius lunatus*. In this report, we present arrays of drops looking like polymer microstructures produced by the two-photon polymerization technique that mimic the micro-ornamentation from the bug's cuticle. A good directionality of oil transport was achieved, directly controlled by the direction of the pointed microstructures at the surface. If the tips of the drop-like microstructures are pointing towards the left side, the liquid front moves to the right and vice versa. Similar effects could be expected for the transport of oily lubricants. These results could, therefore, be interesting for applications in friction and wear reduction.

## 1. Introduction

The interdisciplinary field of biomimetics has been very successful in solving engineering problems by searching for solutions in nature. Through the process of evolution many living organisms developed different structural and chemical material properties that assured the continuation of a certain species.

Technological challenges dealing with wetting and liquid collection and transportation also found solutions in nature. Superhydrophobic and self-cleaning surfaces were inspired by lotus leaves [1]. A highly efficient liquid retention and manipulation can be obtained by artificial open radial fibre arrays mimicking ripe dandelion seeds [2]. Fog collection and moisture harvesting of cacti [3,4], spider silk [4] and Stenocara beetles [5] have served as inspiration for the design and production of surfaces with special geometry and chemistry for wetting and liquid transport. In some cases (e.g. for Texas horned lizards [6,7]), passive liquid transport can be even unidirectional. Passive liquid transport in open triangular-shaped capillary channels is also reported in [8], here unrelated to biomimetic inspiration. Unidirectional fluid transport may be also induced by oriented nano- or microstructures. Experimental [9,10] and theoretical [9] studies on wetting of surfaces covered with bent nanopillars show that a water droplet spreads in a unidirectional manner, along the bent pillars' direction, while it is halted in the opposite direction. Another theoretical study regarding the water transport on a hydrophilic substrate patterned with a triangular post shows that water transport is faster in the direction of the triangles' tips [11].

The Neotropical flat bug *Dysodius lunatus*, a representative of the family *Aradidae*, is widely distributed in South and Central America [12] and lives on and under the bark of various rainforest trees, associated with and feeding on different fungi growing there [13]. *Dysodius lunatus* bugs have two different types of cuticle micro-ornamentation that contribute to fluid transportation. The first one is located on the dorsal surface and represents a web of capillary channels, with the function of immediately spreading water droplets that come in contact with the bug integument. This property provides camouflage, as water changes considerably the reflectance of the bug cuticle (i.e. makes it darker), protecting the bugs from visually oriented predators during rainfall [13]. Little more was known about the fluid transport mechanisms on the cuticle of these bugs as they were rarely studied, even though the bugs were first described by J.C. Fabricius in 1775 [12].

**Figure 1.**
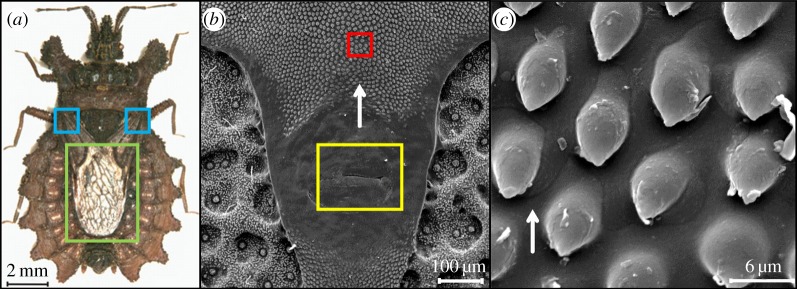
(*a*) Optical image of *Dysodius lunatus* (green rectangle shows the wings and blue squares show the region where the oily defensive liquid is thought to be evaporated). (*b*) SEM micrograph of *D. lunatus*' cuticle under the wing, showing the scent liquid-secreting pore in the yellow square and the surface micro-ornamentation in the red square. (*c*) A higher magnification of the micro-ornamentation region, where the drop-like microstructures can be seen in detail (white arrow shows the preferential flow direction).

We found that *D. lunatus* bugs also possess caudally oriented micro-ornamentation underneath the wings, around the glands that secrete an oily defensive liquid, as an anti-predator adaptation [14]. Even though the gland system is located on the dorsal surface (figure 1*b*), the substance is most likely evaporated at the region of the wings' base (figure 1*a*). We suppose that specifically these microstructures contribute to the transport of the oily substance from the gland system to the wings of the bug (figure 1*c*). These microstructures might also be used for attaching the wings to the body in their folded stage [15]. The micro-ornamentation consists of a periodical array of droplet-like structures of around 10 µm in length, with an undercut. Along the scent gland channel, the microstructures show some variation, as can be seen in the electron micrographs shown in electronic supplementary material, S1. But in all cases, the microstructures have pointed tips which are oriented caudally, i.e. opposite to the bug head. Thus, the tips are pointing to one of the three scent glands beneath. The density and size of the structures are similar over the whole channel length. The wings of *D. lunatus* are kept mostly in the folded state, as these bugs are bark-resting and slow-moving, and show generally a quiescent behaviour [13]. The bug cuticle containing the drop-like structures and the wing together form therefore a closed capillary channel, and the oil transport occurs in the direction from the pointed ends towards the wider ends of these microstructures.

In this study, we are using the micro-ornamentation found around the oil-secreting scent glands underneath the wings of *D. lunatus* as a model for surface patterning to assess oil behaviour in contact with the drop-like microstructure arrays. To produce these bioinspired microstructures at real scale, direct laser writing was used.

## 2. Material and methods

### 2.1. Two-photon lithography set-up

**Figure 2.**
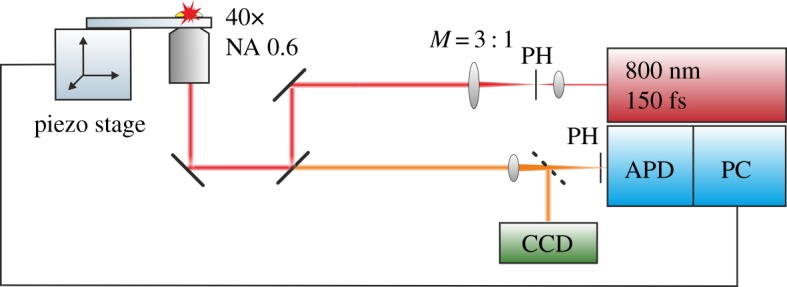
Two-photon lithography set-up (40× = objective lens magnification, NA = numerical aperture of the objective lens, *M* = telescope magnification, PH = pinhole, CCD = charge-coupled device camera, APD = avalanche photodiode, PC = personal computer).

For the fabrication of periodic drop-like microstructures a two-photon polymerization set-up was used [16], as shown in figure 2. The beam of a Ti-sapphire femtosecond laser (Mai Tai, Spectra Physics, 800 nm, 150 fs, 800 MHz) is first mode-cleaned by a 30 µm pinhole (PH), expanded by means of a telescope with a magnification *M* = 1 : 3, and then focused on a glass slide surface by a 40× magnification Olympus objective lens that has a numerical aperture of 0.6. An adjustment ring on the objective lens compensates for the refractive index mismatch between the glass slide and the photoresist. The backscattered light from the glass slide passes through the second dichroic mirror in front of the objective lens and is focused into an avalanche photodiode (APD). Together with the piezo-actuator scanning stages (scan range 1500 × 1500 × 250 µm) and a personal computer (PC), the APD is part of a confocal microscope used to determine the precise position of the air–glass interface, i.e. the starting point of structure-writing. The microstructures were written on a glass substrate (24 × 50 mm^2^) at a speed of 30 µm s^−1^, with a laser excitation power of 21 mW.

### 2.2. Photoresist preparation and function

**Figure 3.**
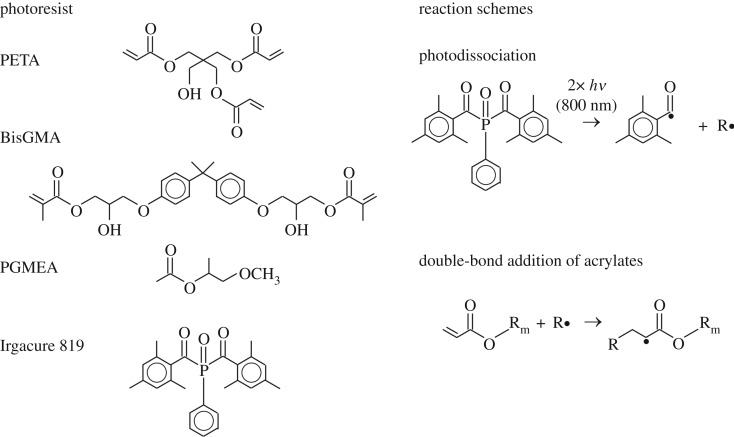
Chemical formulae of the constituents of the photoresist and the reaction schemes of the main steps of the photo-induced polymerization chain reaction.

As a photoresist, an (80/20) mixture of two (meth)acrylate monomers, pentaerythritol triacrylate (PETA, Sigma-Aldrich) and bisphenol A glycidyl methacrylate (BisGMA, Esschem Europe Ltd, Durham, UK), was used. To this mixture 2 wt% Irgacure 819 (BASF, Ludwigshafen, Germany) was added—an efficient photosensitive initiator of the radical polymerization chain reaction in acryl-based compounds. To assure a better mixing of the components and a good fluidity of the final photoresist, 150 ml of propylene glycol methyl ether acetate (PGMEA) was added, and the whole mixture was magnetically stirred for 3 h. The chemical formulae of the constituents of the photoresist are shown on the left side of figure 3.

The right side of figure 3 shows the two main steps of the photo-induced polymerization chain reaction, which is described in detail in [17]. The two-photon absorption of 800 nm photons results in a photodissociation of the photoinitiator (in our case Irgacure 819). The cleaved phosphorus compound or secondary reaction products can then act as radical (R**·**) to start the chain reaction of the (meth)acrylates by double-bond addition.

After the microstructures were written, the un-polymerized photoresist was washed away using the organic solvent Xylol (Chemicals VWR BDH Prolabo, Leicestershire, UK).

### 2.3. Drop-like microstructure design

**Figure 4.**
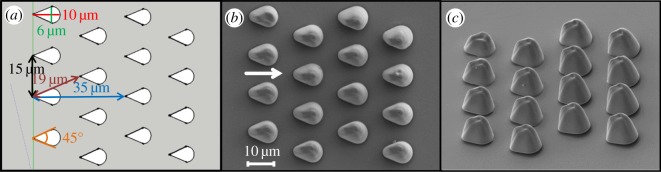
(*a*) 4 × 4 drop-like microstructure array CAD design. (*b*) SEM micrographs of the 4 × 4 drop-like microstructure array, viewed from top (white arrow shows the preferential flow direction) and (*c*) viewed from a tilt angle of 45°.

**Figure 5.**
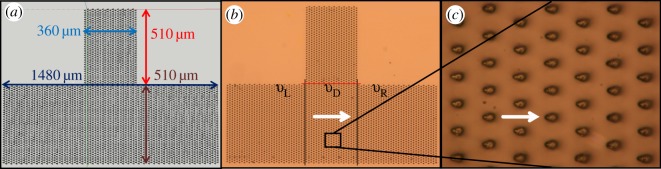
(*a*) The upside-down T array CAD design with the two channels: the red and blue arrows show the width and the length, respectively, of the vertical channel, and the length and the width, respectively, of the horizontal channel. (*b*) Optical microscope image of the upside-down T array and (*c*) higher magnification (white arrow shows the preferential flow direction).

The three-dimensional design of the drop-like microstructure arrays was executed using the SketchUp Make software (figures 4*a* and 5*a*). Further, the computer-aided designs (CADs) were saved in stereolithography format (.stl) and uploaded into a cross-platform program (Keep It Simple Slicer, KISSlicer) that uses .stl files to generate a path code (.gcode) for the piezo-electric stage.

To simplify the research system, the microstructures were designed without undercut, as they naturally occur in our biological example. Each drop-like microstructure has a length of approximately 10 µm, a width of 6 µm and a height of 15 µm (figure 4). The whole design resembles an upside-down T (figure 5*a*,*b*)—the upper vertical channel is used to guide the oil front to the horizontal channel, and the assessment of the directional movement of the oil is done in the latter one. The vertical channel has a width of 360 µm and a length of 510 µm, while the horizontal one has a width of 510 µm and a length of 1480 µm.

### 2.4. Contact angle measurements

To assess the different wetting properties of oil on the glass and photoresist surfaces, contact angle measurements were performed. Two different glass slides were used. Both glass slides were first washed with ethanol and dried, to avoid the presence of any impurities on the surfaces. On one of the glass slides a thin layer of photoresist was spin-coated and subsequently cured with a UV lamp.

For static contact angle measurements the sessile drop method was used, each time 2 µl volume of immersion oil (Immersol 518N, Carl Zeiss AG, Oberkochen) being added. According to the data sheet of the supplier, the oil has a kinematic viscosity of 840 mm^2^ s^−1^ at 23°C. For each sample, five contact angle measurements were performed. The static contact angles were assessed after the drop had settled on a horizontal sample surface. Dynamic measurements were performed using the tilted plate method.

### 2.5. Sample analysis

For the visualization of the structures and video acquisition, a Nikon microscope was used. To assess the behaviour of oil in contact with the microstructures, a droplet of immersion oil was placed in the vicinity of the vertical channel and the oil spreading was recorded. The oil droplet was placed using the tip of a hair 60 µm in diameter. To estimate the volume of the immersion oil droplets, 500 droplets were weighed and the volume was calculated using their density (*ρ* = 0.972 g cm^−3^ at 20°C).

In the natural model, the structures are located under the wings of the bug, and hence to replicate the experiment as close as possible to reality, after the oil droplet was placed near the vertical channel, a round glass coverslip (15 mm diameter) was positioned on the top of both the oil droplet and structure. In this way, the microstructure array written on a glass substrate and the coverslip positioned on the top form together a closed capillary channel.

## 3. Results

The static contact angle measurements of immersion oil revealed a value of 20.4 ± 1.2° on the glass slide and 22.5 ± 0.5° on the photoresist-covered glass slide. These results show that both surfaces are oleophilic, therefore, suitable for our study purpose. From measurements of moving droplets on a tilted plate, we found that the advancing oil contact angles were about 10° higher than the static ones, while the receding oil contact angles were low (i.e. below 10°).

**Figure 6.**
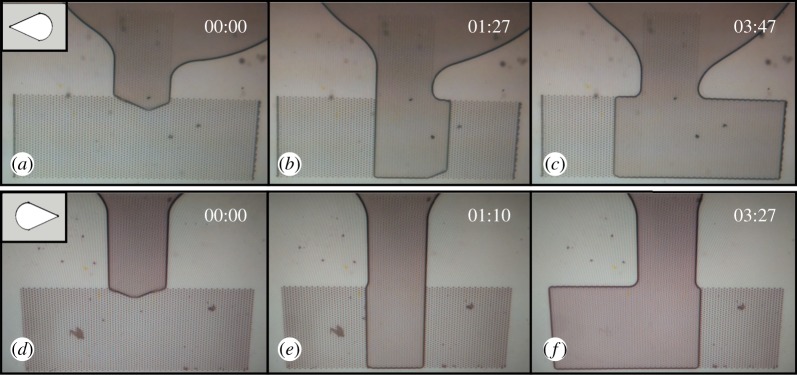
Highlight pictures depicting the oil movement in contact with the array under the cover slide glass; microstructures' tips oriented to the left (*a*–*c*) and to the right (*d*–*f*). Field of view 1 mm× 1.5 mm.

To assess the directionality of the oil transport on the polymer arrays written on a glass substrate, a small amount of oil was positioned on the glass slide, close to the vertical guiding channel. The mean volume of an oil droplet was calculated to be 125.5 ± 28.6 nl. Then a round glass cover slide was placed on both the oil droplet and the array. Multiple videos of the oil front dynamics were recorded. As soon as the cover glass touches the oil droplet and the microstructure array, the oil front starts spreading due to capillary forces. The pressure exerted by the cover slide is estimated to be approximately 4 Pa. Figure 6 highlights the oil dynamics in contact with the array in between the glass slides and the coverslip.

In figure 6*a–c*, the microstructures' tips are oriented towards the left side and the first image (figure 6*a*) depicts the moment when the oil front passes the vertical channel and is guided to the horizontal channel. In figure 6*b*, it can be seen that the oil front reached the bottom rim of the array and started moving towards the right side (in the opposite direction of the microstructures' tips). In 2 min, the oil reaches the right rim of the array, while on the left side it traversed a much smaller distance (figure 6*c*). In order to avoid systematic errors (such as uneven height of the microstructures on the left and right rims and therefore a different pressure distribution), the design was mirror-imaged relative to the *z*-axis, and hence in this design (figure 6*d*–*f*) the drop-like structures' tips are oriented to the right. The same manner of movement was noticed. After the oil front passes through the vertical guiding channel (figure 6*d*), it reaches the bottom rim of the array (figure 6*e*) and then it is clearly seen that the movement is sustained against the tips of the micro-ornamentations to the left side of the array and halted in the opposite direction (figure 6*f*). The full dynamics of the liquid movement can be seen in the videos in electronic supplementary material, S2 and S3.

For comparison, we performed measurements with arrays of microstructures without a coverslip. The oil droplet in this case was placed even closer to the vertical guiding channel than for covered microstructure arrays. Oil behaviour on arrays with no coverslip (microstructures' tips oriented to left and to the right) showed no directional transport. Once the oil front reached the horizontal channel, it started moving in all other three directions—down, left and right, with more or less the same speed, regardless of the microstructures' tips orientation. The oil front reached the arrays' left and right rims almost at the same time (with an average difference of 5 s).

Based on the recorded videos (electronic supplementary material, S2 and S3), three different oil front speeds were calculated: the speed of the oil front down from the horizontal red line (figure 5*b*) to the array's bottom rim, *v*_D_; oil front speed to the left and right sides from the vertical black lines, *v*_L_ and *v*_R_, respectively. Oil front speed in the vertical channel was not taken into consideration, as it decreases considerably when the oil front reaches the vertical channel. For the array with the microstructures pointing to the left side (figure 6*a*–*c*) next values were obtained: *v*_D_ ≈ 5.9 µm s^−1^, *v*_R_ ≈ 4 µm s^−1^ and *v*_L_ ≈ 1.8 µm s^−1^. Similar values for the array with the microstructures pointing to the right (figure 6*d*–*f*) were obtained: *v*_D_ ≈ 7.4 µm s^−1^, *v*_R_ ≈ 4.1 µm s^−1^ and *v*_L_ ≈ 1.9 µm s^−1^. The difference between the *v*_D_ in the two arrays might be attributed to the different oil volume and to the different initial position of the oil droplet relative to the array before putting a coverslip on top. Obviously, *v*_D_ (oil front speed in the perpendicular direction relative to microstructures' orientation) is higher than oil front speeds to the left and right sides of the array. Even so, in both cases, the speed of the oil front against the microstructures' tips is around 2.2 times higher than that in the opposite direction.

In the case of oil movement with no coverslip, the speed of the oil front down to the array's bottom rim, *v*_D_, had a value of 15 µm s^−1^, while the speeds to the right and left rims, *v*_R_ and *v*_L_, had the same value of 13 µm s^−1^. These three similar values prove the absence of a preferential flow direction of the oily liquid.

From all the videos, regardless of the drop-like microstructures’ orientation and the presence or lack of coverslip, it can be noted that the polymer microstructures sustain wetting better than the glass surface, as the oil does not exceed the limits of the array. From these videos it can be clearly concluded that the geometry of the drop-like microstructures makes the oil transport against their tips easier and faster, while their wider region slows down the liquid front.

## 4. Discussion

In this study we have used two-photon lithography to produce bioinspired acrylic polymer microstructure on a glass substrate, and have tested wetting by transport of oil. Even though the oil front has a higher speed when the structure is not covered and when the oil moves perpendicularly towards the microstructures' orientation, we succeeded in obtaining a clear preferential direction of the oil movement (in the horizontal channel) against the tips of the micro-ornamentation when the structures are covered by a glass coverslip.

In the case of a free surface (uncovered), the liquid droplet's height is larger than that of the microstructures. Thus the structure acts as surface roughness, leading to enhanced wetting, i.e. to a uniform spreading of the liquid in all directions. This can be explained by the Wenzel-model for wetting of structured surfaces [18]. In the case of capillary transport (covered), the droplet-like microstructures can be interpreted as distributed defects on a smooth surface. Joanny & de Gennes [19] derived a theory for the pinning of a liquid front, i.e. the hysteresis of the advancing and receding contact angle on a surface containing an ensemble of identical defects distributed over it. They obtained a relationship between the advancing and receding contact angles, *θ*_A_ and *θ*_R_, respectively, to be

4.1 $$\text{cos}\;\theta_{R}-\text{cos}\;\theta_{A}=nW=\frac{nf_{m}^{2}}{2\pi \gamma^{2}}\text{ln}\frac{L}{r},$$

where *n* is the number of defects per unit area; *W* is the total energy dissipated by one defect; *f*_m_ is the maximum force a defect can exert on the triple line before a jump occurs (maximal pinning force), which is dependent on the materials involved; *γ* is the surface tension; *L* is the average distance between adjacent defects; and *r* is the typical dimension (radius) of a defect. In our case, *n*, *f*_m_, *γ* as well as *L* are identical in both directions. However, the defect radii are different.

**Figure 7.**
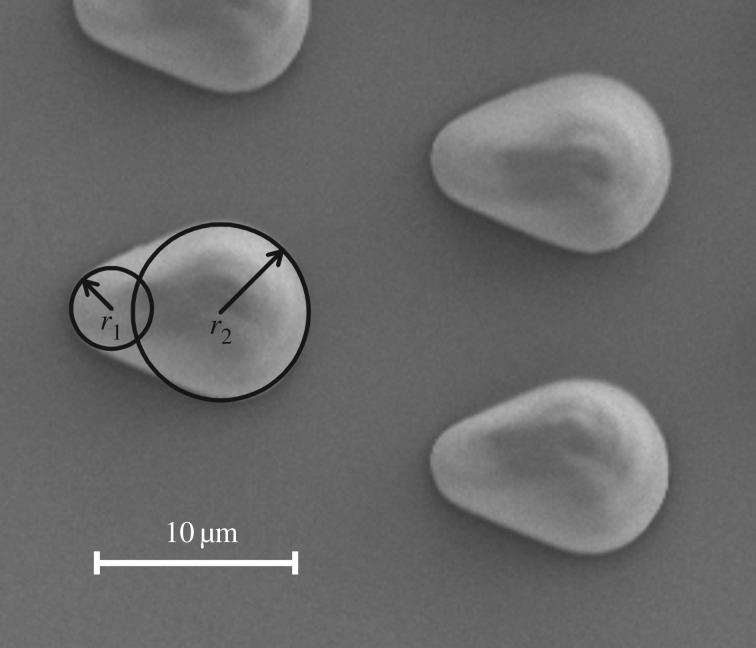
Different radii of the drop-like microstructures as defects on the glass slide surface. The smaller radius *r*_1_ is about 2 µm and the larger radius *r*_2_ is about 4.5 µm.

As can be seen from figure 7, the smaller radius *r*_1_ of our microstructures is about 2 µm, while the larger radius *r*_2_ is about 4.5 µm. The average distance *L* between the defects is approximately 13 µm. The fraction in the above formula is constant for the given material combination, but the logarithm of *L/r* is different for the two transport directions by a factor of approximately 2. Thus, the liquid flows and increases the contact angle at the fronts until the advancing contact angle is reached and then the liquid jumps. While at the larger radius *r*_2_ the advancing contact angle is overcome and the liquid front jumps, the small radius *r*_1_ pins the front better and due to the jump in the direction of *r*_2_, increasing the apparent wetted area, the contact angles are decreased again. This can be observed repetitively until the one side of the structure is completely filled with liquid. Then the flowing liquid can increase the contact angle at the sides of *r*_1_ and also overcome the pinning. When the oil front moves transversally to the microstructures (i.e. down in the figures), it encounters defects with even much larger radii (almost infinite) than both *r*_1_ and *r*_2_. According to the formula, this should result in less pinning making the liquid front move faster, as is observed experimentally.

Interestingly, our system is homogeneous in the sense that the oil contact angles are similar for the substrate and the microstructures. The same applies for oily fluids in contact with micro-patterned or rough surfaces in tribology or microfluidics. Therefore, we anticipate that this research can lead to multiple industrial applications in friction and wear reduction. From our ongoing systematic studies varying the microstructure size and geometry and applying different fluids, we already can say that the effect of directional fluid transport depends on both the viscosity and the contact angle of the fluid. The contact angle should be in the order of around 20° to 40°. Both properties have, on the other hand, to be appropriate for the envisaged technical application.

## Supplementary Material

Supplement 1. SEM micrographs of the scent gland channel of Dysodius lunatus’ cuticle under the wing. In the left column of the figure, several images are stitched together showing the whole channel length with the three liquid secreting pores (indicated by white arrows). The middle and the right column show magnified details with oriented pointed microstructures.

## Data accessibility

The videos of the liquid transport of the microstructures are provided as .mp4 format in the electronic supplementary material.
