# Supplementary figures and images for: Bioinspired polymer microstructures for directional transport of oily liquids
Source: R Soc Open Sci. 2017 Mar 15;4(3):160849. doi: 10.1098/rsos.160849 (PMC5383830; doi:10.1098/rsos.160849)

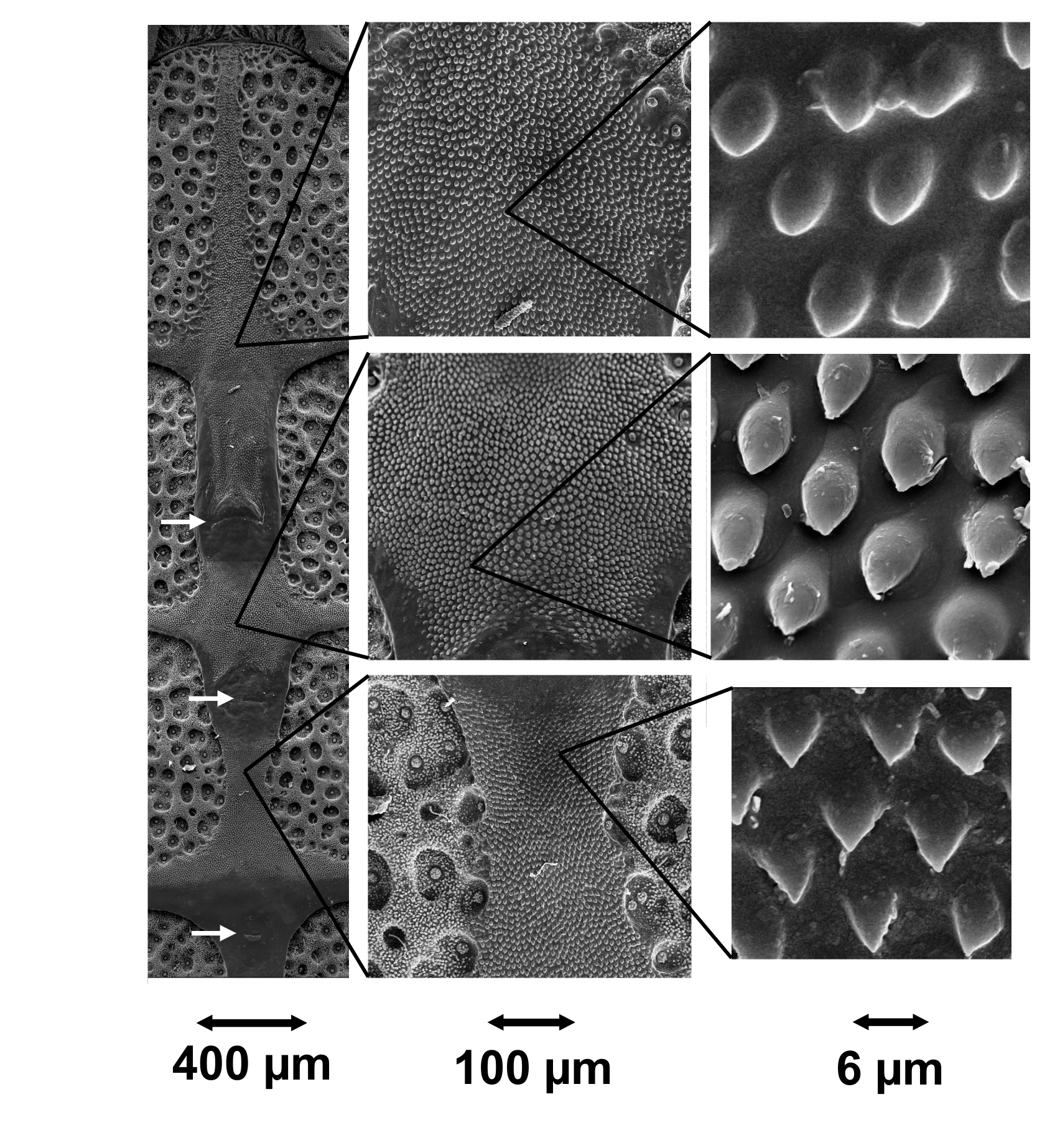

Supplement: Supplement 1. SEM micrographs of the scent gland channel of Dysodius lunatus’ cuticle under the wing. In the left column of the figure, several images are stitched together showing the whole channel length with the three liquid secreting pores (indicated by white arrows). The middle and the right co [file rsos160849supp1.tif]
